# A nationwide survey concerning the mortality and risk of progressing severity due to arterial and venous thromboembolism in inflammatory bowel disease in Japan

**DOI:** 10.1007/s00535-021-01829-5

**Published:** 2021-10-05

**Authors:** Katsuyoshi Ando, Mikihiro Fujiya, Kenji Watanabe, Sakiko Hiraoka, Hisashi Shiga, Shinji Tanaka, Hideki Iijima, Tsunekazu Mizushima, Taku Kobayashi, Masakazu Nagahori, Hiroki Ikeuchi, Shingo Kato, Takehiro Torisu, Kiyonori Kobayashi, Masaaki Higashiyama, Toshiro Fukui, Takashi Kagaya, Motohiro Esaki, Shunichi Yanai, Daiki Abukawa, Makoto Naganuma, Satoshi Motoya, Masayuki Saruta, Shigeki Bamba, Makoto Sasaki, Kazuhiko Uchiyama, Katsuyuki Fukuda, Hideo Suzuki, Hiroshi Nakase, Toshiaki Shimizu, Masahiro Iizuka, Mamoru Watanabe, Yasuo Suzuki, Tadakazu Hisamatsu

**Affiliations:** 1 Gastroenterology and Endoscopy, Division of Metabolism and Biosystemic Science, Gastroenterology, and Hematology/Oncology, Department of Medicine, Asahikawa Medical University, 2-1 Midorigaoka-higashi, Asahikawa, Hokkaido 078-8510 Japan; 2 Center for Inflammatory Bowel Disease, Division of Internal Medicine, Hyogo College of Medicine, Hyogo, Japan; 3 Department of Gastroenterology and Hepatology, Graduate School of Medicine, Dentistry and Pharmaceutical Sciences, Okayama University, Okayama, Japan; 4 Division of Gastroenterology, Tohoku University Graduate School of Medicine, Miyagi, Japan; 5 Department of Endoscopy and Medicine, Hiroshima University Hospital, Hiroshima, Japan; 6 Department of Gastroenterology and Hepatology, Osaka University Graduate School of Medicine, Osaka, Japan; 7 Department of Gastroenterological Surgery and Department of Therapeutics for Inflammatory Bowel Diseases, Graduate School of Medicine, Osaka University, Osaka, Japan; 8 Center for Advanced IBD Research and Treatment, Kitasato University Kitasato Institute Hospital, Tokyo, Japan; 9 Department of Gastroenterology and Hepatology, Tokyo Medical and Dental University, Tokyo, Japan; 10 Department of Inflammatory Bowel Disease, Division of Surgery, Hyogo College of Medicine, Hyogo, Japan; 11 Department of Gastroenterology and Hepatology, Saitama Medical Centre, Saitama Medical University, Saitama, Japan; 12 Department of Medicine and Clinical Science, Graduate School of Medical Sciences, Kyushu University, Fukuoka, Japan; 13 Research and Development Center for New Medical Frontiers, Kitasato University School of Medicine, Kanagawa, Japan; 14 Division of Gastroenterology and Hepatology, Department of Internal Medicine, National Defense Medical College, Saitama, Japan; 15 The Third Department of Internal Medicine, Division of Gastroenterology and Hepatology, Kansai Medical University, Osaka, Japan; 16 Departments of Gastroenterology, National Hospital Organization Kanazawa Medical Center, Kanazawa, Japan; 17 Division of Gastroenterology, Department of Internal Medicine, Faculty of Medicine, Saga University, Saga, Japan; 18 Division of Gastroenterology, Department of Internal Medicine, School of Medicine, Iwate Medical University, Iwate, Japan; 19 Division of General Pediatrics and Gastroenterology, Miyagi Children’s Hospital, Miyagi, Japan; 20 Division of Gastroenterology and Hepatology, School of Medicine, Keio University, Tokyo, Japan; 21 IBD Center, Hokkaido Prefectural Welfare Federation of Agricultural Cooperative, Sapporo-Kosei General Hospital, Hokkaido, Japan; 22 Division of Gastroenterology and Hepatology, Department of Internal Medicine, The Jikei University School of Medicine, Tokyo, Japan; 23 Division of Digestive Endoscopy, Shiga University of Medical Science, Shiga, Japan; 24 Division of Gastroenterology, Department of Internal Medicine, Aichi Medical University School of Medicine, Aichi, Japan; 25 Molecular Gastroenterology and Hepatology, Graduate School of Medical Science, Kyoto Prefectural University of Medicine, Kyoto, Japan; 26 Department of Gastroenterology, St Luke’s International Hospital, Tokyo, Japan; 27 Department of Gastroenterology, Graduate School of Institute Clinical Medicine, University of Tsukuba, Ibaraki, Japan; 28 Department of Gastroenterology and Hepatology, Sapporo Medical University School of Medicine, Hokkaido, Japan; 29 Department of Pediatrics and Adolescent Medicine, Juntendo University Graduate School of Medicine, Tokyo, Japan; 30 Health Care Center, Akita Red Cross Hospital, Akita, Japan; 31 Institute of Innovation Advancement, Tokyo Medical and Dental University, Tokyo, Japan; 32 Department of Internal Medicine, Toho University Medical Center Sakura Hospital, Chiba, Japan; 33 Department of Gastroenterology and Hepatology, Kyorin University School of Medicine, Tokyo, Japan

**Keywords:** Venous thromboembolism, Arterial thromboembolism, Inflammatory bowel disease, Mortality, Severity

## Abstract

**Background:**

The mortality and risk factors of severe disease and death due to arterial and venous thromboembolism (ATE and VTE, respectively) in patients with inflammatory bowel disease (IBD) remain unclear, especially in Asia.

**Aims:**

This study aimed to reveal the mortality and risk factors of TE in IBD patients in Japan.

**Methods:**

In the primary surveillance, responses to questionnaires regarding the number of cases of severe TE and TE-associated death in IBD patients in a span of over the past 10 years were obtained from 32 institutions in Japan. In the secondary surveillance, detailed data about IBD patients with TE were collected. The characteristics, laboratory data, therapy status, and situation at the time of TE development were retrospectively collected, and the data were compared between the patients with and without severe TE and TE-associated death.

**Results:**

The incidence of TE was 1.89% among 31,940 IBD patients. The frequencies of severe TE and TE-associated mortality were 10.7% and 1.0% among the total IBD and TE with IBD patients, respectively. The only risk factor for severe ATE and ATE-associated death was ischemic heart disease. The independent risk factors for severe VTE and VTE-associated death were age (≤ 45 years old), the site of VTE, and disease severity, with anti-TNF therapy as a potential negative risk factor. Patients with severe VTE had a high risk of developing persistent VTE and sequelae.

**Conclusion:**

Unlike ATE, the incidence of VTE was comparable in Asian and Western countries. Therapeutic and prophylactic strategies for managing IBD-associated TE in Asia are urgently needed.

**Supplementary Information:**

The online version contains supplementary material available at 10.1007/s00535-021-01829-5.

## Introduction

Inflammatory bowel disease (IBD) is a chronic and relapsing intestinal and systemic inflammatory disorder that has two main phonotypes: ulcerative colitis (UC) and Crohn’s disease (CD). Extraintestinal manifestations, as well as intestinal complications such as stenosis and abscess formation, frequently occur in IBD. Thromboembolism (TE) is a serious extraintestinal manifestation [1–4] that has been suggested to correlate with chronic inflammation, hypercoagulability, endovascular dysfunction, and atherosclerosis [5, 6]. Indeed, large-scale cohort studies from Western countries have shown that the incidence of venous thromboembolism (VTE) in IBD patients was two- to threefold greater than that in non-IBD controls [7, 8].

In recent years, several reports have suggested that IBD patients have an increased risk of arterial thromboembolism (ATE), including ischemic heart disease (IHD) and cerebrovascular disease (CVD), compared with non-IBD patients [9–11]. Notably, ATE and VTE are regarded as life-threatening complications of IBD in clinical practice due to the potential need for invasive therapy (admission to the intensive care unit, catheter intervention, and surgery) and consequences of severe organ failure and death [1]. In previous studies, the mortality rate of IBD-associated VTE has been reported to range from 10.7% to 25% [12–14]; however, most of these reports included data from before the start of biologics treatment (1970s to 2000s), regions other than Asia and races other than Asians. Nguyen et al. reported that the in-hospital mortality rate of IBD patients with VTE was 2.5- and 2.1-times greater than that in IBD patients without VTE and non-IBD patients with VTE, respectively [15]. Furthermore, in that study, the risk factors of in-hospital death in patients with IBD were suggested to be UC, an older age, presence of comorbidities, health insurance (Medicare or Medicaid), and IBD-related surgery in addition to VTE [15]. However, these risk factors for in-hospital mortality in IBD were analyzed in hospitalized patients who died of IBD, so these were not risk factors for death in IBD patients with VTE. Conversely, according to two meta-analyses, the mortality of IBD patients with ATE was comparable to that of non-IBD controls with ATE [8, 16]. It has also been reported that the mortality of ATE was increased in specific subgroups of IBD; those with colonic-type CD have a risk of cardiovascular disease, women ≥ 40 years old have a risk of myocardial infarction, and women < 40 years old have a risk of stroke and related diseases [17–19]. These data from Western countries clearly show the high incidences of TE and TE-associated disease severity in IBD patients.

In Asian countries, a multi-national study revealed that the incidence of VTE in hospitalized IBD patients was 2.27 times greater than that in non-IBD controls, [20] although the sample size of that study was much smaller than those in studies from Western countries [4, 7]. No large-scale nationwide study concerning the incidence and risk factors for TE-associated severity and death in IBD patients has been reported from Eastern countries, and the incidences and mortalities of ATE and VTE in the general population differ between Eastern and Western countries [21, 22]. Pharmacological prophylaxis for VTE has been recommended for all hospitalized IBD patients in Western countries according to various guidelines and consensus statements [7, 23, 24]; however, in Asia, pharmacological prophylaxis has not been described due to a lack of evidence. Therefore, clarifying the incidence of TE and risk factors of TE-associated severity and death in IBD patients in Eastern countries is necessary to establish an appropriate risk classification of VTE and reasonable strategies for screening and prophylaxis.

The present nationwide surveillance aimed to determine the incidence of TE and risk factors of TE-associated severity and death in an Asian population.

## Methods

### Primary surveillance for the incidence of TE and TE-associated severity and death in IBD patients

A questionnaire concerning the incidence of TE, ATE, VTE, and severe TE and TE-associated death in IBD patients was distributed to participating institutions on the Research Committee of Inflammatory Bowel Disease the Ministry of Health and Welfare of Japan, and 32 institutions responded to the questionnaire. The subjects of the survey were IBD patients who visited the participating institutions from January 2008 to December 2017.

The questionnaire inquired about the following: (1) the total number of IBD patients (UC and CD, respectively) in each institute, (2) the number of IBD patients (UC and CD, respectively) who developed ATE and VTE among the patients in each institute, and (3) the number of IBD patients who developed severe TE and/or TE-associated death among the patients in item 2. The number of severe and non-severe ATE/VTE patients that did and did not result in death was surveyed with a database and chart review in each institution. ATE and VTE were confirmed by imaging, regardless of the presence of symptoms. ATE consisted of the following: CVD, such as cerebral thrombosis and infarction; IHD, such as angina pectoris and acute myocardial infarction; limbs and peripheral ATE; and mesenteric ATE. VTE consisted of the following: deep venous thromboembolism (DVT) in the lower limbs (L), pulmonary arterial thromboembolism (PE) with or without DVT in L, cerebral venous sinus thrombosis (CVS), portal and mesenteric VTE (PMV), and catheter-related thrombosis (CR). Severe TE was defined as cases requiring invasive therapy, including catheter intervention, surgery, and intensive care, and leading to severe organ failure, such as heart, respiratory, liver, and renal failure due to TE.

### Secondary surveillance for the risk factors of severe TE and TE-associated death in IBD patients

The secondary surveillance was performed as a retrospective study to determine the features and risk factors of IBD patients who developed severe TE and TE-associated death. Following the primary surveillance, the details of all IBD cases with ATE and VTE, including those that developed severe TE and death, during the period covered by the primary surveillance were collected by a database and chart review in each institute. The definitions of ATE, VTE, and severe TE were similar to those used in the primary surveillance. The characteristics, activity of IBD, laboratory data and medical therapy for IBD at the time of the development of VTE, site of thrombosis, treatment for ATE and VTE, and the outcomes, including the severity, sequelae, and death due to ATE and VTE, were retrospectively collected in IBD patients who developed ATE or VTE. The data in cases with severe TE and TE-associated death were compared with those in cases without severe TE and TE-associated death.

### Endpoints

The primary endpoint of this study was the incidence of severe TE and TE-associated mortality in IBD patients. The secondary endpoints were as follows: the incidence of severe TE and TE-associated mortality at each site of TE development, the risk factors for TE-associated severity and death in IBD patients, the period from the onset of TE until severe disease and death, therapy and outcomes after developing TE, and hemorrhagic complications of antithrombotic and fibrinolytic therapy.

The study protocol was approved by the ethics committees of Asahikawa Medical University (authorization number 18139) and other participating institutions and was registered with the University Hospital Medical Information Network (UMIN) Center (UMIN registry number 000030524). Informed consent was obtained by announcing this study on the Internet and providing the opportunity to opt out.

### Data collection

The following patient characteristics were collected: age, sex, height, body weight, body mass index (BMI), disease duration, type of IBD (UC or CD), type of disease extension (UC: Montreal classification; E1, 2, 3, CD: Montreal classification; L1, 2, 3, 4), disease activity (UC: partial Mayo score [pMayo], CD: Crohn’s disease activity index [CDAI]; clinical remission was defined as pMayo ≤ 2 or CDAI ≤ 150, mild activity was defined as pMayo 3–5 or CDAI 151–219, and moderate to severe activity was defined as pMayo ≥ 6 or CDAI ≥ 220), extraintestinal manifestations, history of smoking and alcohol consumption, history of thrombosis and thrombotic events, intake of antithrombotic drugs, presence or absence of central venous catheter [CVC], medications (5-aminosalicylate [5-ASA], prednisolone [PSL], immunomodulators [IM], calcineurin inhibitor [CNI], anti-TNF-α antibody, ustekinumab [UST], vedolizumab [VDZ], Janus kinase [JAK] inhibitor, and cytapheresis), history of bowel surgery, systemic comorbidities (diabetes mellitus [DM], hypertension [HT], dyslipidemia [DLp], heart and pulmonary disease, renal dysfunction, neuromuscular disease, and malignancy), and presence or absence of a long-term bedridden status (defined as performance status ≥ 2).

Data on the following conditions at the time of ATE and VTE development and therapy toward ATE and VTE were also collected: ambulatory or hospitalized at the time of ATE or VTE development, hospitalization period until the development of ATE or VTE, site of ATE (CVD, IHD, limbs and peripheral ATE, mesenteric ATE, and others), site of VTE (DVT in L, pulmonary ATE, CVS, PVM, catheter-related thrombosis, and others), presence or absence of TE-associated severity or death, period until development of severe disease or death, therapy for ATE and VTE (antithrombotic therapy, fibrinolytic therapy, inferior vena cava filter, catheter intervention, surgery, physical therapy, or observation only), presence or absence of hemorrhagic complications of antithrombotic and fibrinolytic therapy, and outcomes after therapy (improvement with elimination or retention of thrombosis, retention of sequelae, death, or uncertain).

The following laboratory data were also recorded at admission or before the onset of TE (data just before diagnosing TE): white blood cell and platelet counts, serum levels of hemoglobin, C-reactive protein, total protein, albumin, blood urea nitrogen, total cholesterol, triglyceride, and hemoglobin A1c, erythrocyte sedimentation rate (per hour), fibrinogen level, antithrombin III (AT-III), international normalized ratio of prothrombin time (PT-INR), active partial thromboplastin time (APTT), D-dimer, fibrin degradation product (FDP), and thrombin-antithrombin complex (TAT).

### Statistical analyses

All statistical analyses were performed using the SPSS software program. Fisher’s exact test was used to compare the incidence of ATE and VTE between two or more groups. The incidence and proportion among three or more groups were compared using Fisher’s exact test with Holm’s correction. An unpaired *t*-test and Fisher’s exact test were used to compare patient characteristics and laboratory data between IBD patients with and without severe disease and death. The cutoff values of the items with significant differences were determined by a receiver operating characteristics analysis. A logistic regression analysis was used to identify the independent risk factors leading to severe disease and death in IBD with ATE and VTE, which were detected by entering the significant factors revealed in the univariate analysis into a multivariate model. A *p* value < 0.05 was considered statistically significant.

## Results

### Primary surveillance

A total of 31,940 IBD patients (21,186 and 10,754 with UC and CD, respectively) who visited the 32 participating institutions within 10 years (2008–2017) were enrolled in this study. The number of IBD patients who developed TE was 604 (1.89%), which consisted of 374 (1.77%) and 195 (1.81%) patients with UC and CD, respectively. The annual incidence rate of TE was 188.8 per 100,000 person-years in IBD, 176.5 per person-year in UC, and 181.3 per person-year in CD. The number of ATE patients with IBD was 278 (0.87%), which consisted of 175 (0.83%) and 103 (0.96%) patients with UC and CD, respectively. In addition, the number of VTE patients with IBD was 328 (1.03%), which consisted of 202 (0.95%) and 91 (0.84%) patients with UC and CD, respectively. The incidence of ATE and VTE in IBD patients was almost equal, as was that between UC and CD. The number of patients who developed severe TE and/or TE-associated death was 65 (0.20% of all IBD patients and 10.7% of IBD patients with ATE and VTE). The number of patients with TE-associated death was six (0.019% of all IBD patients and 1.0% of IBD patients with TE). All six patients had UC (0.028% of all UC patients and 1.6% of UC patients who developed TE; among these, two had ATE (0.0094% of all UC patients and 1.14% of UC patients with ATE) and four had VTE (0.019% of all UC patients and 1.98% of UC patients with VTE). Among all IBD patients, the estimated annual rate of severe TE and/or TE-associated death was 20.3 per 100,000 IBD patient-years; the estimated annual mortality due to TE was calculated as 1.88 (0.63 with ATE and 1.25 with VTE), whereas it was 2.83 among UC patients (0.94 with ATE and 1.89 with VTE) per 100,000 patient-years (Table 1).

**Table 1:** Number and incidence of IBD patients with TE and severe TE and TE-associated death in the first nationwide surveillance

	IBD total	UC	CD
Number of total patients	31,940	21,186	10,754
Number of patients developing TE (N,%)	604 (1.89%)	374 (1.77%)	195 (1.81%)
Number of patients developing ATE (N,%)	278 (0.87%)	175 (0.83%)	103 (0.96%)
Number of patients occurring VTE (N,%)	328 (1.03%)	202 (0.95%)	91 (0.84%)
Number of patients with severe TE and/or TE-associated death	65 (0.20%*) (10.7%**)	–	–
Number of patients with TE-associated death	6 (0.019%*) (1.0%**)	6 (0.028%*) (1.6%**)	0
Number of patients with ATE-associated death	2 (0.0063%*) (0.72%**)	2 (0.0094%*) (1.14%**)	0
Number of patients with VTE-associated death	4 (0.012%*) (1.22%**)	4 (0.019%*) (1.98%**)	0
Estimated annual TE-associated mortality (persons per 100,000 IBD patient-year)	1.88	2.83	0

^*^proportion to total IBD patients enrolled in this study

^**^ The proportion to total IBD patients with thromboembolism

*TE* thromboembolism, *ATE* arterial thromboembolism, *VTE* venous thromboembolism

### Secondary surveillance

The detailed data of 215 IBD patients with ATE (n = 48) and VTE (n = 167) during the period of the primary surveillance were collected and analyzed in the secondary surveillance.

### ATE

A total of 48 IBD patients who developed ATE were examined. The demographics of the patients with IBD are shown in Table 2. The average age at onset of ATE was 56.3 years old, and 83.3% (*n* = 40) of the patients with ATE were classified as A3 under the Montreal classification. The proportion of males (*n* = 33, 68.8%) was about twice as high as that of females (*n* = 15, 31.2%). The proportion of UC cases (*n* = 41, 85.4%) was higher than that of CD (*n* = 7, 14.6%), and pancolitis-type (E3) UC accounted for 73.1% of all UC cases. The average disease duration until the development of ATE was 124.2 months. The proportion of patients who were current (*n* = 17) or previous (*n* = 5) smokers was 45.8%. The proportion of patients with comorbidities was 62.5% (n = 30). Most of the patients had cardiovascular risk factors/metabolic diseases (DM: 22.9%, HT: 27.1%, DLp: 16.7%, current or a history of cardio- or CVD: 25.0%).

**Table 2:** Characteristics of IBD patients who developed ATE and VTE

	ATE *N* = 48	VTE *N* = 167
Background of IBD		
Age at the onset (years)	56.3 ± 15.1	47.3 ± 18.4
A1/A2/A3	1/7/40	6/54/107
Gender (M/F)	33/15	88/79
Height (cm)	162.2 ± 13.8	164.4 ± 8.5
BW (kg)	59.2 ± 13.6	55.8 ± 12.2
BMI (kg/m^2^)	22.1 ± 3.6	20.6 ± 4.06
Type of IBD		
UC (*N*,%)	41(85.4%)	120 (71.2%)
CD (*N*,%)	7(14.6%)	47 (28.8%)
Type of UC (E1/E2/E3)	2/9/30	4/19/94
Type of CD (L1/L2/L3)	4/2/1	11/2/33
Disease duration until TE (months)	124.2 ± 160.2	117.1 ± 128.2
EIM (*N*,%)	1 (2.1%)	22 (13.2%)
Lifestyle habit and comorbidity at the onset of TE		
History of smoking (*N*, %)	22 (45.8%)	45 (26.9%)
History of alcohol drinking (*N*,%)	14 (29.2%)	35 (21.9%)
History of VTE (*N*,%)	8 (16.7%)	12 (7.2%)
Comorbidity	30 (62.5%)	73 (43.7%)
Malignancy	1 (2.1%)	13 (7.8%)
DM	11 (22.9%)	8 ( 4.7%)
HT	13 (27.1%)	13 (7.8%)
DLp	8 (16.7%)	5 (3.0%)
Renal dysfunction	4 (8.3%)	5 (3.0%)
Cardiovascular disease	8 (16.7%)	8 (4.8%)
Cerebrovascular disease	2 (4.2%)	0 (0%)
Neuromuscular disease	0 (0%)	3 (1.8%)
Disease with thrombotic tendency	0 (0%)	4 (2.4%)
Detailed situation at onset of TE		
Medical situation at onset of TE		
Ambulatory (*N*,%)	37 (77.1%)	47 (28.1%)
Hospitalization (*N*,%)	11 (27.9%)	120 (71.9%)
Hospitalization until onset of TE (days)	21.0 ± 19.6	20.3 ± 20.8
Clinical activity at onset of TE		
Partial Mayo (UC)	2.7 ± 2.4	4.6 ± 2.9
CDAI (CD)		239.3 ± 79.1
Patients with moderate to severe IBD activity (*N*,%)	4 ( 8.3%)	55 (32.9%)
Antithrombotic drugs before onset of TE (*N*,%)	6 (12.5%)	3 (1.8%)
Concomitant treatment for IBD		
5-ASA (*N*,%)	34 (70.8%)	109 ( 65.3%)
Corticosteroid (*N*,%)	12 (25.0%)	88 (52.7%)
Immunomodulator (*N*,%)	13 (27.1%)	33 (19.8%)
Calcineurin inhibitor (*N*,%)	1 (2.1%)	10 (6.0%)
Anti-TNF antibody (*N*,%)	5 (10.4%)	28 (16.7%)
Anti-IL12/23 antibody (*N*,%)	0 (0%)	1 (0.6%)
JAK inhibitor (*N*,%)	2 (4.2%)	1 (0.6%)
Vedolizumab (*N*,%)	1 (2.1%)	0 ( 0%)
Cytapheresis (*N*,%)	1 (2.1%)	16 (9.6%)
Central venous catheter (*N*,%)	3 (6.3%)	80 (48.0%)
Bowel resection (*N*,%)	4 (8.3%)	52 (31.3%)
Laboratory data before onset of TE		
WBC (/μl)	7321 ± 3679	8112 ± 4273
Hb (g/dl)	12.2 ± 2.1	11.2 ± 2.2
Ht (%)	37.4 ± 6.2	34.2 ± 6.4
Plt (× 10^6^/μl)	48.4 ± 90.3	32.0 ± 15.8
T-P (g/dl)	6.5 ± 1.0	6.8 ± 5.4
Alb (g/dl)	3.5 ± 0.8	3.1 ± 0.8
BUN mg/dl)	14.9 ± 5.3	11.6 ± 6.3
Cre (mg/dl)	0.95 ± 0.80	0.82 ± 0.87
CRP (mg/dl)	2.53 ± 4.0	3.09 ± 4.4
ESR (mm/hour)	38.3 ± 24.6	36.8 ± 26.1
PT-INR	1.12 ± 0.19	1.42 ± 2.88
APTT (sec)	34.6 ± 9.3	37.6 ± 56.6
Fib (mg/dl)	358 ± 110	371.8 ± 113.4
D-dimer (ng/dl)	10.5 ± 28.3	7.9 ± 21.3
FDP (ng/dl)	52.5 ± 98.8	24.6 ± 52.5
AT-III (%)	91.8 ± 20.8	90.4 ± 20.1

*BW* body weight, *BMI* body mass index, *IBD* inflammatory bowel disease, *ATE* arterial thromboembolism *VTE* venous thromboembolism, *UC* ulcerative colitis, *CD* Crohn’s disease, *EIM* extra-intestinal manifestation, *DM* diabetes mellitus, *HT* hypertension, *DLp* dyslipidemia, *5-ASA* 5-amynosalicylate, *TNF* tumor necrosis factor, *IL* interleukin, *JAK* Janus kinase, *WBC* White blood cell count, *Hb* hemoglobin, *Ht* hematocrit, *Plt* platelet, *T-P* total protein, *Alb* albumin, *BUN* blood urea nitrogen, *Cre* creatinine, *CRP* C-reactive protein, *ESR* erythrocyte sedimentation rate, *PT-INR* prothrombin international rate, *APTT* activated partial thrombin time, *Fib* fibrinogen, *FDP* fibrin degradation product, *AT-III* antithrombin-III

The proportion of patients with ATE who were ambulatory (*n* = 37, 77.1%) was higher than that of patients who were hospitalized (*n* = 11, 27.9%). The average hospitalization period in patients who developed ATE was 21 days. The average pMayo score in patients with UC was 2.7, and the proportion of patients with moderate to severe clinical activity was 8.3% (*n* = 4). The treatment used for IBD was 5-ASA in 34 patients (70.8%), corticosteroids in 12 (25.0%), IM in 13 (27.1%), CNI in 1 (2.1%), anti-TNF antibody in 5 (10.4%), JAK inhibitor in 2 (4.2%), VDZ in 1 (2.1%), cytapheresis in 1 (2.1%), CVC in 3 (6.3%), and bowel resection in 4 (8.3%).

**Fig. 1:**
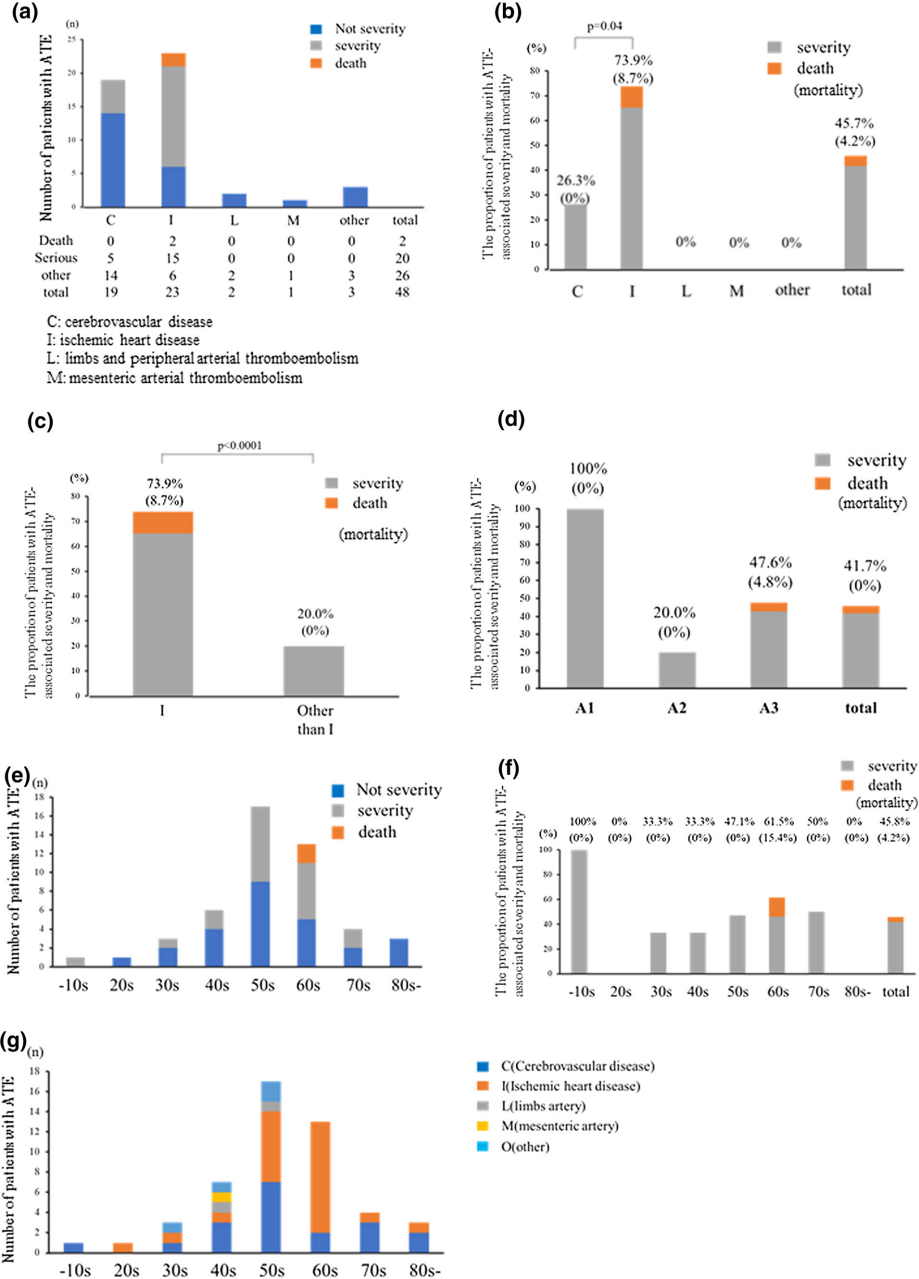
The incidence of severe ATE and ATE-associated death in IBD patients. Each figure shows **a** the number of patients with severe ATE and ATE-associated death at each site, **b** the proportion of severe ATE and ATE-associated death at each site and **c** when classified as IHD versus others, **d** the proportion of severe ATE and ATE-associated death when classified into three groups based on the Montreal classification, **e** the number of cases of severe ATE and ATE-associated death by age (every decade), **f** the proportion of severe ATE and ATE-associated death by age (every decade), and **g** the incidence of ATE at each site and for each age group

The laboratory data before the onset of ATE are shown in Table 2. The number of ATE instances at each site is shown in Fig. 1a. CVD (*n* = 19, 39.6%) and IHD (*n* = 23, 43.8%) accounted for the majority of ATE instances, in contrast to two cases (4.2%) in the limbs and peripheral ATE, one case of mesenteric ATE, and three cases at other sites. The total number of patients who developed severe ATE and ATE-associated death was 20 (41.7%) and 2 (4.2%), respectively, in the cohort collected at the secondary surveillance (Fig. 1b). The proportion of severe ATE and ATE-associated death in IHD (73.9%, with death accounting for 8.7% [*n* = 2]) was significantly higher than that in CVD (26.3%, with death accounting for 0%) (*p* = 0.040). The proportion of severe ATE and ATE-associated death in IHD patients (73.9%) was also much higher than that in non-IHD patients (20%) (*p* < 0.0001) **(**Fig. 1c**)**. Regarding the age, the proportion of severe ATE and ATE-associated death in patients classified as A1, A2, and A3 was 100% (1/1), 20% (1/5), and 50.5% (20/42, mortality 4.8%), respectively (Fig. 1d). Furthermore, when analyzing age groups by decade, the proportion of severe ATE and ATE-associated death in patients in their ≤ 10 s, 20 s, 30 s, 40 s, 50 s, 60 s, 70 s, and ≥ 80 s was 100% (1/1), 0% (0/1), 33.3% (1/3), 33.3% (2/6), 52.9% (8/17), 61.5% (8/13), 50% (2/4), and 0% (0/3), respectively (Fig. 1e). The proportion of severe ATE and ATE-associated death ranged from 30 to 60% in patients in their 30 s to 70 s, with the incidence of ATE peaking in patients in their 50 s and 60 s; both patients who died due to ATE were in their 60 s (Fig. 1f). Regarding the development site and age, CVD peaked in patients in their 50 s and sporadically occurred in any age. In addition, IHD peaked in patients in their 50 s and 60 s and rarely occurred in other age groups (Fig. 1g).

To identify the risk factors for severe ATE and ATE-associated death, the patients’ characteristics, therapy for IBD, and laboratory data before the onset of ATE were compared between the groups with and without severe ATE and ATE-associated death. In the univariate and multivariate analyses, the only risk factor for severe ATE and ATE-associated death was the site of development of ATE, i.e., IHD (77.2% in IHD vs. 23.1% at other sites; odds ratio [OR]: 66.7, 95% confidence interval [CI]: 5.37–793.7, *p* = 0.0010), as summarized in Table 3a and 3b. All variables included in the univariate analysis are described in Table S1.

**Table 3:** Univariate and multivariate analyses comparing the characteristics between groups with and without severe ATE and ATE-associated death.

Univariate analysis	Severity and death (*n* = 22)	Other (*n* = 26)	*p* value
Age at the onset (years)	55.6 ± 15.5	57.0 ± 15.4	0.757
Male (*N*,%)	17 (19.3%)	15 (19.0%)	1
BW (kg)	55.9 ± 14.3	62.1 ± 12.6	0.119
BMI (kg/m2)	21.7 ± 3.5	22.5 ± 3.6	0.439
Type of IBD			1
UC (*N*,%)	19 (86.4%)	22 (79.2%)	
CD (*N*,%)	3 (13.6%)	4 (84.6%)	
Disease duration until ATE (months)	81.7 ± 108.3	157.5 ± 186.9	0.135
History of smoking (*N*, %)	11 (50.0%)	11 (42.3%)	0.772
Comorbidity	14 (63.6%)	16 (61.5%)	1
Malignancy	0 (0%)	1 (3.8%)	1
DM	3 (13.6%)	8 (30.8%)	0.189
HT	5 (22.7%)	8 (30.7%)	0.746
DLp	4 (18.1%)	4 (15.4%)	1
Renal dysfunction	0 (0%)	4 (15.4%)	0.114
Cardiovascular disease	4 (18.1%)	4 (15.4%)	1
Cerebrovascular disease	2 (9.1%)	0 (0%)	0.205
Type of ATE CVD/IHD/L/M/other	5/17/0/0/0	14/6/2/1/3	0.0041
Proportion of IHD	17 (77.2%)	6 (23.1%)	0.00038
Hospitalization at onset of ATE	5 ( 22.7%)	17 (23.1%)	1
Hospitalization until onset of ATE (days)	28.8 ± 27.5	14.8 ± 9.6	0.320
Clinical activity at onset of ATE			
Partial Mayo (UC)	2.5 ± 2.2	2.2 ± 1.9	0.698
Patients with moderate to severe IBD activity (*N*,%)	3(13.6%)	1(3.8%)	0.320
Antithrombotic drugs before onset of ATE (*N*,%)	4 ( 18.2%)	2 (7.7%)	0.484
Concomitant usage of treatment for IBD			
Corticosteroid (*N*,%)	4 (18.2%)	8 (30.8%)	0.495
Immunomodulator (*N*,%)	5 (30.7%)	8 (30.8%)	0.739
Anti-TNF antibody (*N*,%)	2 (9.1%)	3 (11.5%)	1
Central venous catheter (*N*,%)	3 (13.6%)	0 (0%)	0.084
Bowel resection (*N*,%)	1 (4.5%)	3 (11.5%)	0.613
Laboratory data before onset of ATE			
WBC (/μl)	7516 ± 3937	7196 ± 3595	0.804
Hb (g/dl)	12.7 ± 1.7	12.0 ± 2.4	0.391
Plt (× 10^6^/μl)	37.8 ± 60.5	55.2 ± 105.9	0.578
Alb (g/dl)	3.7 ± 0.68	3.4 ± 0.89	0.367
Cre (mg/dl)	0.77 ± 0.21	1.06 ± 0.99	0.286
CRP (mg/dl)	2.97 ± 4.7	2.23 ± 3.5	0.597
D-dimer (ng/dl)	17.1 ± 38.6	2.65 ± 3.9	0.430
FDP (ng/dl)	62.6 ± 111.1	12.0 ± 23.5	0.711
AT-III (%)	95.5 ± 22.1	77.0 ± 25.4	0.508

Multivariate analysis		
Variables	OR (95% CI)	*p* value
BW	0.99(0.86–1.12)	0.824
Comorbidity-DM	5.08(0.38–66.2)	0.21
Comorbidity-Renal dysfunction		0.997
Site of ATE: IHD	66.7(5.37–793.7)	0.0010
Central venous catheter		0.997

*BW* body weight, *BMI* body mass index, *IBD* inflammatory bowel disease, *VTE* venous thromboembolism, *UC* ulcerative colitis, *CD* Crohn’s disease, *DM* diabetes mellitus, *HT* hypertension, *DLp* dyslipidemia, *CVD* cerebrovascular disease, *IHD* ischemic heart disease, *L* limbs and peripheral arterial thromboembolism, *M* mesenteric arterial thromboembolism, 5-ASA: 5-amynosalicylate, *TNF* tumor necrosis factor, *WBC* White blood cell count, *Hb* hemoglobin, *Plt* platelet, *Alb* albumin, *BUN* blood urea nitrogen, *Cre* creatinine, *CRP* C-reactive protein, *FDP* fibrin degradation product, *AT-III* antithrombin-III, *CI* confidence interval, *OR* odds ratio

Therapy for ATE consisted of antithrombotic therapy in 31 patients (64.5%), fibrinolytic therapy in 8 (16.7%), catheter intervention in 12 (25.0%), and surgery in 4 (8.3%); none received observation without therapy. The proportion of patients who received antithrombotic and fibrinolytic therapy did not significantly differ between the groups with and without severe ATE and ATE-associated death. Hemorrhagic complications due to antithrombotic or fibrinolytic therapy arose in two cases (4.2%), both in the group with severe ATE and ATE-associated death (Table S3).

The outcomes after ATE are described in Table S4. The average duration until a severe condition developed from the onset of ATE was 1.1 days. The duration until death from the onset of ATE was 0 and 320 days in the two cases that died. The outcomes after ATE consisted of improvement in 29 (60.4%), retention of sequelae in 10 (20.8%), death in 2 (4.2%), and uncertain in 7 (14.6%). The improvement rate in the group with severe ATE and ATE-associated death (40.9%) tended to be lower than in the group without it (76.9%), while the retention rate of sequelae (31.8%) in the group with severe ATE and ATE-associated death tended to be higher than in the group without it (11.5%).

### VTE

A total of 167 IBD patients who developed VTE were examined. The demographics of the patients with IBD are shown in Table 2. The average of age at the onset of ATE was 47.3 years old, and the Montreal classification was A1 in 6 (3.6%), A2 in 54 (32.3%), and A3 in 107 (64.7%). The proportion of males (*n* = 88, 52.7%) was comparable to that of females (*n* = 79, 47.3%). The average BMI was 20.6 kg/m^2^. The proportion of UC cases (*n* = 120, 71.2%) tended to be higher than that of CD (*n* = 47, 28.8%), and pancolitis-type (E3) UC accounted for 77.5% of all UC cases, whereas L3 of CD accounted for 70.2% of all CD cases. The average disease duration until the development of VTE was 117.1 months. The proportion of patients who were current or previous smokers was 28.8% and 7.2%, respectively. Regarding comorbidities, 7.8% of patients (*n* = 13) had malignancy, 4.7% (*n* = 8) had DM, 7.8% (*n* = 13) had HT, 3.0% (*n* = 5) had DLp, 3.0% (*n* = 5) had renal dysfunction, 4.8% (*n* = 8) had cardiovascular disease, 1.8% (*n* = 3) had neuromuscular disease, and 2.4% (*n* = 4) had a thrombotic tendency.

The proportion of patients with VTE who were hospitalized (*n* = 120, 71.9%) was higher than that of patients who were ambulatory (*n* = 47, 28.1%), which was the opposite of the tendency seen in cases with ATE. The average hospitalization period in patients who developed VTE was 20.3 days. The average pMayo score and CDAI in patients with UC and CD were 4.6 and 239.3, respectively, and the proportion of patients with moderate to severe clinical activity was 32.9% (*n* = 55), suggesting that the clinical activity in patients with VTE tended to be higher than in those with ATE. The treatment used for IBD was 5-ASA in 109 patients (65.3%), corticosteroids in 88 (52.7%), IM in 33 (19.8%), CNI in 10 (6.0%), anti-TNF antibody in 28 (16.7%), anti-IL12/23 antibody in 1 (0.6%), JAK inhibitor in 1 (0.6%), plasmacytapheresis in 16 (9.6%), CVC in 80 (48.0%), and bowel resection in 52 (31.3%); VDZ was not used by any patient. Of note, the proportion of VTE patients treated with corticosteroids, CVC, and bowel resection tended to be higher than that of ATE patients (vs. 25.0%, 6.3%, and 8.3%, respectively).

**Fig. 2:**
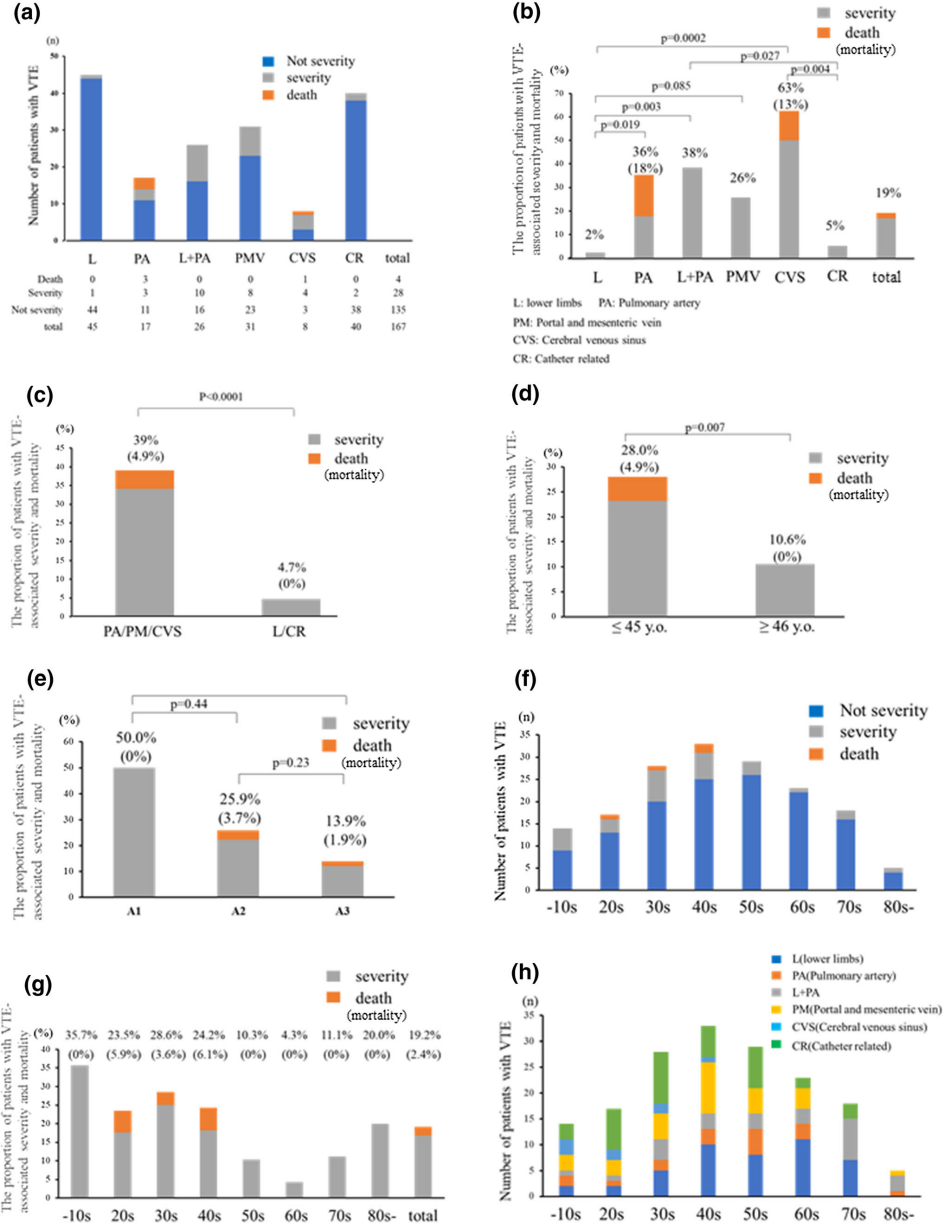
The incidence of severe VTE and VTE-associated death in IBD patients. Each figure shows **a** the number of patients with severe VTE and VTE-associated death in each developing site, **b** the proportion of severe VTE and VTE-associated death in each developing site and **c** when classified with IHD and the others, **d** the proportion of severe VTE and VTE-associated death when classified with two groups in a boarder of age 45 and **e** three groups based on Montreal classification, **f** number of severe VTE and VTE-associated death in each age (every 10 years), **g** the proportion of severe VTE and VTE-associated death in each age (every 10 years), and **h** the incidence of VTE at each site and for each age group

Laboratory data before the onset of VTE are shown in Table 2. The number of VTE instances at each site among the 167 cases is shown in Fig. 2a. These patients had DVT in L (*n* = 45, 26.9%), PA (*n* = 17, 10.2%), DVT in L and PA (*n* = 26, 15.6%), PMV (*n* = 31, 18.6%), CVS (*n* = 8, 4.8%), and CR (*n* = 40, 24.0%). The proportion of VTE-associated severity and death at each site is shown in Fig. 2b. The proportion of patients who developed severe VTE and VTE-associated death was 17.0% (*n* = 32; death: 2.4%) in the cohort of the secondary surveillance. The proportion of severe VTE and VTE-associated death was 2.0% (*n* = 1) in DVT in L, 36.0% (*n* = 6) in PA, 38.0% (*n* = 10) in DVT in L and PA, 26.0% (*n* = 8) in PMV, 63.0% (*n* = 5) in CVS, and 5.0% (*n* = 2) in CR. The proportions of severe VTE and VTE-associated death in L + PA (38.0%) and CVS (63.0%) were significantly higher than those of L (2.0%) and CR (5.0%) (*p* = 0.003 in L + PA vs. L, *p* = 0.027 in L + PA vs. CR, *p* = 0.0002 in CVS vs. L, *p* = 0.004 in CVS vs. CR). The proportion of severe VTE and VTE-associated death in PA (36%) was significantly higher than that in L (*p* = 0.019), and that in PMV (26.0%) tended to be higher than that in L (*p* = 0.085). The mortality rate due to VTE was 18.0% (*n* = 3) and 13.0% (*n* = 3) in PA and CVS, respectively, and no deaths occurred in cases concerning L, L + PA, PMV, PMV, and CR. The proportion of severe VTE and VTE-associated death in PA/PM/CVS (34.1%; mortality, 4.9%) was much higher than that at other sites (4.7%) (*p* < 0.0001) (Fig. 2c). The proportion of severe VTE and VTE-associated death in patients ≤ 45 years old (28.0%) was significantly higher than that in patients ≥ 46 years old (10.6%, *p* = 0.007). Notably, death due to VTE occurred only in patients ≤ 45 years old (mortality: 4.9%) (Fig. 2d). Regarding the Montreal classification, the proportions of severe VTE and VTE-associated death of 50.0% (*n* = 3), 25.9% (*n* = 14), and 13.9% (*n* = 15) in A1, A2, and A3, respectively, showed no significant difference, although the proportions gradually increased with decreasing age (Fig. 2e). Furthermore, on analyzing the age groups by decade, the incidence of VTE peaked in patients in their 40 s among all generations. The proportion of severe VTE and VTE-associated death in patients in their ≤ 10 s, 20 s, 30 s, 40 s, 50 s, 60 s, 70 s and ≥ 80 s was 35.7% (5/14), 23.4% (4/17), 28.6% (8/28), 24.2% (8/33), 10.3% (3/29), 4.3% (1/23), 11.1% (2/18), and 20.0% (1/5), respectively. The proportion of severe VTE and VTE-associated death in patients in their 10 s to 40 s tended to be higher than that in patients in their 50 s to 70 s, while the incidence of VTE in patients in their 50 s to 70 s was comparable to that in patients in their 10 s to 40 s (Fig. 2f, g). Regarding the development site and age, CVS predominantly occurred in patients in their 10 s to 40 s, while the incidence of L and/or PA gradually increased with age (Fig. 2h).

To identify the risk factors for severe VTE and VTE-associated death, the patients’ characteristics, therapy for IBD, and laboratory data before the onset of VTE were compared between the groups with and without severe VTE and VTE-associated death. In the univariate analysis, the risk factors for severe VTE and VTE-associated death were a young age (≤ 45 years old), no history of smoking, site of development of VTE (PA, PMV, CVS), moderate to severe disease activity, non-use of anti-TNF antibody, and an elevated CRP level before the onset of VTE (≥ 1.5 mg/dL) (Table 4a). In the multivariate analysis, the independent risk factors for severe VTE and VTE-associated death were age ≤ 45 years old (OR: 2.84, 95% CI: 1.05–7.69, *p* = 0.038), the site of development of VTE (PA, PMV and CVS) (OR: 9.79, 95% CI: 3.03–31.6, *p* = 0.00013), and disease severity (moderate to severe) (OR: 2.79, 95% CI: 1.04–7.47, *p* = 0.0415) (Table 4b). All variables included in the univariate analysis are described in Table S2.

Therapy for VTE consisted of antithrombotic therapy in 140 patients (83.8%), fibrinolytic therapy in 13 (7.8%), IVC filter in 11 (6.6%), catheter intervention (other than an IVC filter) in 9 (5.4%), surgery in 8 (4.8%), and observation without therapy in 20 (12.0%). Fibrinolytic therapy in the group with severe VTE and VTE-associated death (*n* = 6, 18.8%) was performed more frequently than in the group without it (*n* = 7, 5.2%), and there was no untreated case in the group of severe VTE and VTE-associated death. In addition, 15.0% of patients without severe VTE and VTE-associated death were untreated. A significant difference was observed in the incidence of untreated cases between the two groups (*p* = 0.015) (Table S5).

Hemorrhagic complications due to antithrombotic or fibrinolytic therapy arose in nine cases (5.4%), and the proportion of hemorrhagic complications in the group with severe VTE and VTE-associated death (16.1%) was significantly higher than in the group without it (3.0%) (*p* = 0.012) **(Table S5)**.

The outcomes after VTE are described in Table 5. The average duration until a severe condition developed from the onset of VTE was 3.3 days. The average duration until death from the onset of VTE was 13.5 days. VTE was resolved and remained in 95 (56.9%) and 52 (31.4%) patients, respectively, sequelae remained in 6 (3.6%) patients, 4 (2.4%) patients died, while the outcomes in 10 (6.0%) patients were uncertain. In the group with severe VTE and VTE-associated death, VTE was resolved and sequelae remained in nine (40.9%) and seven (31.8%) patients, respectively. The proportion with sequelae in the group of severe VTE and VTE-associated death (12.5%) tended to be higher than in the group without severe VTE and VTE-associated death (0%), while the proportions with improvement regardless of the elimination of VTE were comparable between the groups (75.0% vs. 85.9%).

**Table 4:** Univariate and multivariate analyses comparing the characteristics between groups with and without severe VTE and VTE-associated death

Univariate analysis			
	Severity and death (*n* = 32)	Other (*n* = 135)	*p* value
Age at the onset (years)	40.1 ± 17.5	49.1 ± 18.2	0.013
A1/A2/A3	3/14/15	3/40/92	0.030
Age ≤ 45 years (N,%)	23 (28.0%)	9 (10.6%)	0.0056
Male (N,%)	17 (19.3%)	15 (19.0%)	1
BMI (kg/m2)	21.5 ± 4.7	20.4 ± 3.9	0.180
Type of IBD			0.513
UC/CD(N,%)	25 (20.8%)/7 (14.9%)	95(79.2%)/40 (85.1%)	
Type of UC (E1/E2/E3)	3/3/19	1/16/75	0.182
Type of CD (L1/L2/L3)	2/0/4	9/2/29	0.581
Disease duration until VTE (months)	86.8 ± 109.6	124.0 ± 131.5	0.153
History of smoking (*N*, %)	3 (10%)	42 (31.1%)	0.045
History of VTE (*N*,%)	3 (9.3%)	9 (6.7%)	0.702
Comorbidity			
Malignancy	1 (3.1%)	12 (8.8%)	0.466
DM	1 (3.1%)	7 ( 5.2%)	1
HT	3 (9.3%)	10 (7.4)	0.716
DLp	1 (3.1%)	4 (3.0%)	1
Renal dysfunction	0 (0%)	5 (3.7%)	0.584
Cardiovascular disease	2 (6.2%)	6 (4.4%)	0.650
Site of VTE: L/PA/L + PA/PM/CVS/CR/	1/6/10/8/5/2	44/11/16/23/3/38	< 0.0001
Proportion of PA/PM/CVS	28 (87.5%)	54 (40.0%)	< 0.0001
Situation at onset of VTE – hospitalization	24 (75.0%)	96 (71.1%)	0.827
Hospitalization until onset of VTE (days)	22.4 ± 35.9	18.2 ± 14.5	0.381
Clinical activity at onset of VTE			
Partial Mayo (UC)	5.4 ± 3.2	3.2 ± 2.9	0.0019
CDAI (CD)	257.0 ± 14.1	227.6 ± 97.7	0.620
Patients with moderate to severe IBD activity (*N*,%)	16 (50.0%)	39 ( 29.5%)	0.0079
Antithrombotic drugs before onset of VTE (*N*,%)	1 (3.1%)	2 (1.6%)	1
Concomitant usage of treatment for IBD			
5-ASA (*N*,%)	22 (68.8%)	87 (64.4%)	0.686
Corticosteroid (*N*,%)	21 (65.6%)	67 (49.6%)	0.118
Immunomodulator (*N*,%)	5 (15.6%)	28 (20.7%)	0.626
Calcineurin inhibitor (*N*,%)	1 (3.7%)	9 (6.6%)	0.686
Anti-TNF antibody (*N*,%)	1 (3.1%)	27 (20.0%)	0.018
Central venous catheter (*N*,%)	15 (46.8%)	65 (48.1%)	1
Bowel resection (*N*,%)	8 (25.0%)	44 (32.6%)	0.525
Laboratory data before onset of VTE			
WBC (/μl)	9113 ± 5189	7866 ± 4003	0.153
Hb (g/dl)	10.9 ± 1.93	11.2 ± 2.27	0.451
Plt (× 10^6^/μl)	30.3 ± 20.0	32.4 ± 14.6	0.508
Alb (g/dl)	3.1 ± 0.83	3.2 ± 0.83	0.559
CRP (mg/dl)	4.99 ± 5.97	2.60 ± 3.84	0.008
CRP ≥ 1.5 mg/dl (*N*,%)	19 (59.4%)	49 (36.3%)	0.0266
D-dimer (ng/dl)	9.06 ± 15.6	7.71 ± 22.5	0.844
FDP (ng/dl)	30.0 ± 48.1	23.0 ± 54.5	0.727
AT-III (%)	88.1 ± 21.5	91.6 ± 19.7	0.639

Multivariate analysis		
Variables	OR (95% CI)	*p* value
Age at onset ≤ 45 years	2.84 (1.05–7.69)	0.038
History of smoking	0.337(0.085–1.34)	0.122
Site of VTE: PA/PM/CVS	9.79 (3.03–31.6)	0.00013
Moderate to severe IBD activity	2.79(1.04–7.47)	0.0415
Usage of anti-TNF antibody	0.119 (0.014–1.05)	0.055
CRP before onset of VTE ≥ 1.5 mg/dl	2.13 (0.78–5.26)	0.148

*BW* body weight, *BMI* body mass index, *IBD* inflammatory bowel disease, *VTE* venous thromboembolism, *UC* ulcerative colitis, *CD* Crohn’s disease, *DM* diabetes mellitus, *HT* hypertension, *DLp* dyslipidemia, *L* deep venous thromboembolism in lower limbs, *PE* pulmonary arterial thromboembolism, *CVS* cerebral venous sinus thrombosis, *PVM* portal and mesenteric venous thromboembolism, *CR* catheter-related thrombosis, *5-ASA* 5-amynosalicylate, *TNF* tumor necrosis factor, *IL* interleukin, *JAK* Janus kinase, *WBC* White blood cell count, *Hb* hemoglobin, *Plt* platelet, *Alb* albumin, *CRP* C-reactive protein, *FDP* fibrin degradation product, *AT-III* antithrombin-III, *CI* confidence interval, *OR* odds ratio

**Table 5:** The outcomes after developing VTE

Days until a serious condition developed from the onset of VTE (days)	3.3 ± 6.3
Days until death from onset of VTE (days)	13.5 ± 13.3

Outcome of therapy for VTE	Total (*n* = 167)	Severity (*n* = 32)	Other (*n* = 135)
improvement with elimination of VTE	95 (56.9%)	17 (53.1%)	78 (57.8%)
improvement with retention of VTE	52 (31.4%)	7 (21.9%)	45 (28.1%)
retention of sequelae	6 (3.6%)	4 (12.5%)	2 (1.5%)
Death	4 (2.4%)	4 (12.5%)	0 (0%)
Uncertain	10 (6.0%)	0 (0%)	10 (7.4%)

## Discussion

To the best of our knowledge, the present study is the first nationwide cohort study to describe the incidence and risk factors of severe TE and TE-associated death in IBD patients in Japan and the largest such study in Asia (questionnaire based on patients’ background characteristics). In the first large cohort surveillance (including over 30,000 IBD patients across 10 years), the incidence of TE in IBD patients was 1.89% (188.8 per 100,000 IBD person-years). In addition, the incidence of TE between patients with UC (1.77%, 176.5 per 100,000 UC person-years) and CD (1.81%, 181.3 per person-year) was comparable. Regarding ATE in the present study, the incidence and mortality rate in Japanese IBD patients was 0.87% (86.9 per 100,000 IBD person-years) and 4.2% per IBD-associated ATE patients. The age-standardized incidence rate of stroke and coronary artery disease in the Japanese general population (1988–2000) was reported to be 1,446 per 100,000 person-years in a large cohort study [25], suggesting that Japanese IBD patients are unlikely to have an increased risk of ATE compared to non-IBD patients. In contrast, according to a meta-analysis by Sighn et al., the incidence of ATE (including all IHD, CVD, and peripheral ATE) in Western IBD patients was 5.92% (9,050 of 152,756 IBD patients)^11^, which was higher than in our study. Several recent reports have suggested that IBD patients in Western countries had a 1.18- to 8.07-fold higher risk of ATE, including IHD and CVD, than non-IBD patients [9–11]. These findings suggest that the incidence of IBD-associated ATE markedly differs between Asian and Western countries. The low incidence and mortality rate of ATE in Asian IBD patients might be associated with the different incidence of ATE in the general population between Asian and Western countries [26]. Regarding VTE in the present study, the incidence in IBD was 1.03% (102.5 per 100,000 IBD person-years) in Japan. Weng et al. reported that the incidence of VTE was 0.9% in the hospitalized cohort and 115 per 100,000 person-years in an East Asian cohort and that the incidence was comparable between UC and CD [20]. This showed that the incidence of VTE was almost 100 per 100,000 IBD person-years, which was much higher than that in the general Asian population [27, 28]. In Western countries, the incidence of VTE was reported to be 74.5 to 314 per 100,000 person-years (14 to 30 in DVT and 10 to 20, and 13 to 31 and 10 to 11 in UC and CD patients, respectively) [4, 29–31]. Unlike ATE, the incidence of IBD-associated VTE in Asian countries [32–36] was not lower than that in Western countries. While ATE is known to develop in association with arterial sclerosis, VTE is thought to develop due to the excessive activation of inflammation- and coagulation-related molecules [37, 38]. The different pathogenesis might lead to the different incidences between ATE and VTE in Asian IBD patients.

On comparing the characteristics between the patients with ATE and VTE, regardless of TE-associated severity or death, VTE patients tended to develop at a younger age and have a lower BMI, lower rate of a smoking history and comorbidities (especially DM, HT, CVD), higher clinical activity and higher rates of hospitalization (average pMayo in UC 4.6 ± 2.9 vs. 2.7 ± 2.4 and the proportion of moderate to severe activity 32.9% vs. 8.3%), concomitant use of corticosteroids, and a central venous catheter and bowel resection at the onset of TE than ATE patients (Table 2). These findings suggest that a higher disease activity and rate of thrombogenesis-related medical intervention (corticosteroid, catheter indwelling, and surgery) were potential risk factors in VTE with IBD, whereas common risk factors for TE, such as obesity, lifestyle, and comorbidities (DM, HT, and cardiovascular disease), might be more strongly associated with the onset of ATE with IBD. This discrepancy in the characteristics between ATE and VTE may have contributed to the differences in the incidences of ATE and VTE between IBD patients and the general population.

In this study, the incidence of severe TE and TE-associated death with IBD was 0.20% among all IBD patients and 10.7% among IBD patients with TE. The mortality of IBD patients with TE was 0.019% among all IBD patients (annual mortality due to TE: 1.88 per 100,000 person-years) and 1.0% among IBD patients with TE, which was not higher than that in general population. Regarding ATE, a meta-analysis in Western countries showed that the mortality of cardiovascular disease in IBD patients was not higher than that in the general population [8], although the risk of ATE in IBD patients has been reported to be higher than that in non-IBD controls [9–11]. These findings suggest that the incidences of severe ATE and ATE-associated death in IBD patients were not higher than those in the general population in Asian or Western countries. Regarding VTE in the present study, the mortality of VTE with IBD was 0.012% among all IBD patients and 1.22% among IBD patients with VTE. In previous studies from Western and Latin American countries, VTE-associated mortality in IBD was reported to range from 10.7 to 25% [12–15]. Nguyen et al. reported that the risk of in-hospital mortality in IBD patients with VTE was 2.5- and 2.1-fold higher than that in IBD patients without VTE and non-IBD patients with VTE, respectively [15]. Recently, Weng et al. reported that the in-hospital mortality of VTE was 8.3% (2/24) among patients in an East Asian cohort based on a small sample size [20]. It is difficult to precisely determine the differences in mortality rates between Asian and Western countries due to the differences in study designs and sample sizes. Conversely, to our knowledge, no data on the incidence or outcomes of severe VTE have been published in Asian or Western countries. Our results indicate that the incidence of severe VTE was almost 20%, with certain cases having higher risks of persistent VTE, sequelae, and death. This suggests that aggressive screening is needed to detect VTE before its severity progresses to improve the prognosis of IBD patients with VTE.

In cases of ATE, the proportion of severity and death among IHD patients was markedly higher than that among patients with CVD or other ATE. Our multivariate analysis revealed that IHD was the only independent factor associated with severe ATE and ATE-associated death in IBD patients. A recent study from Western countries showed that a higher disease activity carried a potential risk of ATE [39, 40]; however, in our study, the clinical activity at the onset of ATE in the group with severe ATE and ATE-associated death and the average pMayo value of 2.5 were comparable to those in the group without severe ATE and ATE-associated death. The differences in the physical condition and prevalence of comorbidities (including traditional risk factors for CVD and IHD) between Asian and Western populations might have to be taken into consideration.

The multivariate analysis in this study found that the risk factors for severe VTE and VTE-associated death with IBD were a young age (≤ 45 years old), the site of VTE (PA, PMV, and CVS), and the disease severity (moderate to severe). CVS is known to be associated with the highest risk of severity and death in young patients. Elevated proportions of pulmonary artery embolism and PMV have been demonstrated in young patients. These findings indicate the high risk of severity and death in young IBD patients with PA, PMV, and CVS, although the incidence of IBD-associated TE is low in the younger population. Our study also showed that a high disease activity was a positive risk factor, whereas the usage of anti-TNF antibody might be a negative one for severe VTE and VTE-associated death. A strong inflammatory condition is associated with hypercoagulation status due to increases in coagulation-associated mediators, including factors V, VII, and VIII, lipoprotein (a), and fibrinogen, and decreases in antithrombin III, protein C/protein S, and tissue factor pathway inhibitor and the platelet activity [37]. TNF-α is known to induce the downregulation of endothelial protein C receptors on thin capillary endothelial cells [38]. A recent French cohort study also showed that the usage of anti-TNF antibody was associated with a reduction in the risk of acute arterial events [39]. These findings clearly indicate that the disease activity is closely associated with the risk of VTE in IBD patients. In this study, very few patients who were administered biologics other than anti-TNF antibody were included, as the study period was set before 2017 when biologics other than anti-TNF antibody were not yet clinically available. Therefore, the efficacy of biologics other than anti-TNF antibody for reducing the rate of TE-associated severity and death was not examined in our study despite being expected due to the comparable clinical effectiveness to anti-TNF antibody. In the future, the effects of various agents, including antibody formulations and JAK inhibitors, on the incidence of TE and TE-associated severity and death should be evaluated.

Several limitations associated with this nationwide cohort study warrant mention. First, the demographics of the total IBD patients at the primary surveillance were lacking because this study aims to clarify the incidence and risk factors of severe TE and TE-associated death in IBD patients. Second, about 65% of the IBD patients with ATE and VTE who had been included in the primary surveillance were not included in the secondary surveillance. However, this study was the first of its kind to clarify the actual state of both ATE and VTE in an Asian population and is the largest nationwide cohort study conducted to date, including over 30,000 IBD patients from referral IBD centers and general hospitals in Japan. Third, this survey targeted high-volume institutions and referral centers for IBD participating in the Research Committee of Inflammatory Bowel Disease the Ministry of Health and Welfare of Japan. Therefore, the study population enrolled in our study might have been biased to some degree, as more refractory and intractable patients with IBD may have been included than is typically encountered in most clinical settings in Japan. A further large-scale survey that includes municipal hospitals and clinics should be considered in the future.

In conclusion, this is the largest nationwide cohort study in Asia to describe the incidences of ATE and VTE and severe cases with death. The risk factors for severity and death were found to be the site of development of ATE and VTE, age, and disease activity. In addition, the therapeutic strategies and outcomes of ATE and VTE in IBD patients were described in this study. Although the incidence of ATE in Asian IBD patients was lower than that in reports from Western countries, the incidence of VTE was comparable between Asian and Western countries. Furthermore, the incidence of severe VTE was 20%, with cases tending to show high risks of persistent VTE, developing sequelae, and dying. Therefore, an adequate risk classification system, screening, and prophylactic strategies for IBD-associated TE in Asian populations are urgently needed.

## Supplementary Information

Below is the link to the electronic supplementary material.

Supplementary file1 (DOCX 40 KB)

## Data Availability

The data underlying this article will be shared on reasonable request to the corresponding author.
