# Supplementary material for: A nationwide survey concerning the mortality and risk of progressing severity due to arterial and venous thromboembolism in inflammatory bowel disease in Japan
Source: J Gastroenterol. 2021 Oct 5;56(12):1062–79. doi: 10.1007/s00535-021-01829-5 (PMC8604847; doi:10.1007/s00535-021-01829-5)
Supplement: Supplementary file 1 — Supplementary file1 (DOCX 40 KB) [file 535_2021_1829_MOESM1_ESM.docx]

**Supplemental materials**

**Table S1. Univariate and multivariate analyses comparing the characteristics between groups with and withou**t **severe ATE and ATE-associated death.**

|  | Univariate analysis | | | Multivariate analysis | |
| --- | --- | --- | --- | --- | --- |
|  | Severity and death(n=22) | Other(n=26) | p-value | OR(95% CI) | p-value |
| Age at the onset (years) | 55.6±15.5 | 57.0±15.4 | 0.757 |  |  |
| A1/A2/A3 | 1/1/20 | 0/4/22 | 0.4293 |  |  |
| Male (N,%) | 17 (19.3%) | 15 (19.0%) | 1 |  |  |
| BW (kg) | 55.9±14.3 | 62.1±12.6 | 0.119 | 0.99(0.86-1.12) | 0.824 |
| BMI (kg/m2) | 21.7±3.5 | 22.5±3.6 | 0.439 |  |  |
| Type of IBD |  |  | 1 |  |  |
| UC (N,%) | 19 (86.4%) | 22 (79.2%) |  |  |  |
| CD (N,%) | 3 (13.6%) | 4 (84.6%) |  |  |  |
| Type of UC (E1/E2/E3) | 1/5/13 | 1/4/17 | 0.850 |  |  |
| Type of CD (L1/L2/L3) | 2/1/0 | 2/1/1 | 1 |  |  |
| Disease duration until ATE (months) | 81.7±108.3 | 157.5±186.9 | 0.135 |  |  |
| EIM (N,%) | 1 (4.5%) | 0 (0%) | 0.458 |  |  |
| History of smoking (N, %) | 11 (50.0%) | 11 (42.3%) | 0.772 |  |  |
| History of alcohol drinking (N,%) | 7 (31.8%) | 7 (26.9%) | 1 |  |  |
| History of VTE (N,%) | 4 (18.1%) | 4 (15.4%) | 1 |  |  |
| Comorbidity | 14 (63.6%) | 16 (61.5%) | 1 |  |  |
| Malignancy | 0 (0%) | 1 (3.8%) | 1 |  |  |
| DM | 3 (13.6%) | 8 (30.8%) | 0.189 | 5.08(0.38-66.2) | 0.21 |
| HT | 5 (22.7%) | 8 (30.7 % ) | 0.746 |  |  |
| DLp | 4 (18.1%) | 4 (15.4%) | 1 |  |  |
| Renal dysfunction | 0 (0%) | 4 (15.4%) | 0.114 |  | 0.997 |
| Cardiovascular disease | 4 (18.1%) | 4 (15.4%) | 1 |  |  |
| Cerebrovascular disease | 2 (9.1%) | 0 (0%) | 0.205 |  |  |
| Neuromuscular disease | 0 (0%) | 0 (0%) | 1 |  |  |
| Disease with thrombotic tendency | 0 (0%) | 0 (0%) | 1 |  |  |
| CVD/IHD/L/M/other | 5/17/0/0/0 | 14/6/2/1/3 | 0.0041 |  |  |
| Proportion of IHD | 17 (77.2%) | 6 (23.1%) | 0.00038 | 66.7(5.37-793.7) | 0.0010 |
| Situation at onset of ATE – hospitalization | 5 ( 22.7%) | 17 (23.1%) | 1 |  |  |
| Hospitalization until onset of ATE (days) | 28.8±27.5 | 14.8±9.6 | 0.320 |  |  |
| Clinical activity at onset of ATE |  |  |  |  |  |
| Partial Mayo (UC) | 2.5±2.2 | 2.2±1.9 | 0.698 |  |  |
| CDAI (CD) |  |  |  |  |  |
| Patients with moderate to severe IBD activity (N,%) | 3( 13.6%) | 1(3.8%) | 0.320 |  |  |
| Antithrombotic drugs before onset of ATE (N,%) | 4 ( 18.2%) | 2 (7.7%) | 0.484 |  |  |
| Concomitant usage of treatment for IBD |  |  |  |  |  |
| 5-ASA (N,%) | 15 (68.2%) | 19 (73.1%) | 1 |  |  |
| Corticosteroid (N,%) | 4 (18.2%) | 8 (30.8%) | 0.495 |  |  |
| Immunomodulator (N,%) | 5 (30.7%) | 8 (30.8%) | 0.739 |  |  |
| Calcineurin inhibitor (N,%) | 0 (0%) | 1 (3.8%) | 1 |  |  |
| anti-TNF antibody (N,%) | 2 (9.1%) | 3 (11.5%) | 1 |  |  |
| anti-IL12/23 antibody (N,%) | 0 (0%) | 0 (0%) | 1 |  |  |
| JAK inhibitor (N,%) | 1 (4.5%) | 1 (3.8%) | 1 |  |  |
| Vedolizumab (N,%) | 1 (4.5%) | 0 (0%) | 0.452 |  |  |
| Cytapheresis (N,%) | 1 (4.5%) | 0 (0%) | 0.452 |  |  |
| Central venous catheter (N,%) | 3 (13.6%) | 0 (0%) | 0.084 |  | 0.997 |
| Bowel resection (N,%) | 1 (4.5%) | 3 (11.5%) | 0.613 |  |  |
| Laboratory data before onset of ATE |  |  |  |  |  |
| WBC (/μl) | 7516±3937 | 7196±3595 | 0.804 |  |  |
| Hb (g/dl) | 12.7±1.7 | 12.0±2.4 | 0.391 |  |  |
| Ht (%) | 38.0±4.9 | 37.2±7.0 | 0.696 |  |  |
| Plt (x10^6^/μl) | 37.8±60.5 | 55.2±105.9 | 0.578 |  |  |
| T-P (g/dl) | 6.6±0.87 | 6.5±1.1 | 0.711 |  |  |
| Alb (g/dl) | 3.7±0.68 | 3.4±0.89 | 0.367 |  |  |
| BUN mg/dl) | 14.7±4.7 | 15.0±5.7 | 0.912 |  |  |
| Cre (mg/dl) | 0.77±0.21 | 1.06±0.99 | 0.286 |  |  |
| CRP (mg/dl) | 2.97±4.7 | 2.23±3.5 | 0.597 |  |  |
| ESR (mm/hour) | 38.4±21.5 | 38.2±29.0 | 0.992 |  |  |
| PT-INR | 1.20±0.26 | 1.06±0.08 | 0.110 |  |  |
| APTT (sec) | 34.0±12.8 | 35.5±9.2 | 0.760 |  |  |
| Fib (mg/dl) | 292.8±98.7 | 445.0±98.7 | 0.0582 |  |  |
| D-dimer (ng/dl) | 17.1±38.6 | 2.65±3.9 | 0.430 |  |  |
| FDP (ng/dl) | 62.6±111.1 | 12.0±23.5 | 0.711 |  |  |
| AT-III (%) | 95.5±22.1 | 77.0±25.4 | 0.508 |  |  |

BW: body weight, BMI: body mass index, IBD: inflammatory bowel disease, VTE: venous thromboembolism, UC: ulcerative colitis, CD: Crohn’s disease, EIM: extra-intestinal manifestation, DM: diabetes mellitus, HT: hypertension, DLp: dyslipidemia, CI: confidence interval, OR: odds ratio, VTE: venous thromboembolism, 5-ASA: 5-amynosalicylate, TNF: tumor necrosis factor, IL: interleukin, JAK: Janus kinase, CI: confidence interval, OR: odds ratio, WBC: White blood cell count, Hb: hemoglobin, Ht: hematocrit, Plt: platelet, T-P: total protein, Alb: albumin, BUN: blood urea nitrogen, Cre: creatinine, CRP: C-reactive protein, ESR: erythrocyte sedimentation rate, PT-INR: prothrombin international rate, APTT: activated partial thrombin time, Fib: fibrinogen, FDP: fibrin degradation product, AT-III: antithrombin-III

**Table S2. Univariate and multivariate analyses comparing the characteristics between groups with and without severe VTE and VTE-associated death.**

|  | Univariate analysis | | | Multivariate analysis | |
| --- | --- | --- | --- | --- | --- |
|  | Severity and death(n=32) | Other(n=135) | p-value | OR(95% CI) | p-value |
| Age at the onset (years) | 40.1±17.5 | 49.1±18.2 | 0.013 |  |  |
| A1/A2/A3 | 3/14/15 | 3/40/92 | 0.030 |  |  |
| Age ≤45 years (N,%) | 23 (28.0%) | 9 (10.6%) | 0.0056 | 2.84 (1.05-7.69) | 0.038 |
| Male (N,%) | 17 (19.3%) | 15 (19.0%) | 1 |  |  |
| Height (cm) | 164.0±8.1 | 164.5±8.6 | 0.772 |  |  |
| BW (kg) | 55.7±1.5 | 55.3±12.1 | 0.326 |  |  |
| BMI (kg/m2) | 21.5±4.7 | 20.4±3.9 | 0.180 |  |  |
| Type of IBD |  |  | 0.513 |  |  |
| UC (N,%) | 25 (20.8%) | 95 (79.2%) |  |  |  |
| CD (N,%) | 7 (14.9%) | 40 (85.1%) |  |  |  |
| Type of UC (E1/E2/E3) | 3/3/19 | 1/16/75 | 0.182 |  |  |
| Type of CD (L1/L2/L3) | 2/0/4 | 9/2/29 | 0.581 |  |  |
| Disease duration until VTE (months) | 86.8±109.6 | 124.0±131.5 | 0.153 |  |  |
| EIM (N,%) | 3 (10.0%) | 19 (14.2%) | 0.769 |  |  |
| History of smoking (N, %) | 3 (10%) | 42 (31.1%) | 0.045 | 0.337(0.085-1.34) | 0.122 |
| History of alcohol drinking (N,%) | 29 (13.8%) | 31 (23.7%) | 0.324 |  |  |
| History of VTE (N,%) | 3 (9.3%) | 9 (6.7%) | 0.702 |  |  |
| Comorbidity |  |  |  |  |  |
| Malignancy | 1 (3.1%) | 12 (8.8%) | 0.466 |  |  |
| DM | 1 (3.1%) | 7 ( 5.2%) | 1 |  |  |
| HT | 3 (9.3%) | 10 (7.4 ) | 0.716 |  |  |
| DLp | 1 (3.1%) | 4 (3.0%) | 1 |  |  |
| Renal dysfunction | 0 (0%) | 5 (3.7%) | 0.584 |  |  |
| Cardiovascular disease | 2 (6.2%) | 6 (4.4%) | 0.650 |  |  |
| Neuromuscular disease | 1 (3.1%) | 2 (1.5%) | 0.474 |  |  |
| Disease with thrombotic tendency | 2 (6.2%) | 2 (1.5%) | 0.166 |  |  |
| L/PA/L+PA/PM/CVS/CR/ | 1/6/10/8/5/2 | 44/11/16/23/3/38 | <0.0001 |  |  |
| Proportion of PA/PM/CVS | 28 (87.5%) | 54 (40.0%) | <0.0001 | 9.79 (3.03-31.6) | 0.00013 |
| Situation at onset of VTE – hospitalization | 24 (75.0%) | 96 (71.1%) | 0.827 |  |  |
| Hospitalization until onset of VTE (days) | 22.4±35.9 | 18.2±14.5 | 0.381 |  |  |
| Clinical activity at onset of VTE |  |  |  |  |  |
| Partial Mayo (UC) | 5.4±3.2 | 3.2±2.9 | 0.0019 |  |  |
| CDAI (CD) | 257.0±14.1 | 227.6±97.7 | 0.620 |  |  |
| Patients with moderate to severe IBD activity (N,%) | 16 (50.0%) | 39 ( 29.5%) | 0.0079 | 2.79(1.04-7.47) | 0.0415 |
| Antithrombotic drugs before onset of VTE (N,%) | 1 ( 3.1%) | 2 (1.6%) | 1 |  |  |
| Concomitant usage of treatment for IBD |  |  |  |  |  |
| 5-ASA (N,%) | 22 (68.8%) | 87 (64.4%) | 0.686 |  |  |
| Corticosteroid (N,%) | 21 (65.6%) | 67 (49.6%) | 0.118 |  |  |
| Immunomodulator (N,%) | 5 (15.6%) | 28 (20.7%) | 0.626 |  |  |
| Calcineurin inhibitor (N,%) | 1 (3.7%) | 9 (6.6%) | 0.686 |  |  |
| anti-TNF antibody (N,%) | 1 (3.1%) | 27 (20.0%) | 0.018 | 0.119 (0.014-1.05) | 0.055 |
| anti-IL12/23 antibody (N,%) | 0 (0%) | 1 (0.74%) | 1 |  |  |
| JAK inhibitor (N,%) | 0 (0%) | 1 (0.74%) | 1 |  |  |
| Vedolizumab (N,%) | 0 (0%) | 0 (0%) | - |  |  |
| Cytapheresis (N,%) | 4 (12.5%) | 12 (8.9%) | 0.512 |  |  |
| Central venous catheter (N,%) | 15 (46.8%) | 65 (48.1%) | 1 |  |  |
| Bowel resection (N,%) | 8 (25.0%) | 44 (32.6%) | 0.525 |  |  |
| Laboratory data before onset of VTE |  |  |  |  |  |
| WBC (/μl) | 9113±5189 | 7866±4003 | 0.153 |  |  |
| Hb (g/dl) | 10.9±1.93 | 11.2±2.27 | 0.451 |  |  |
| Ht (%) | 33.7±5.15 | 34.4±6.65 | 0.626 |  |  |
| Plt (x10^6^/μl) | 30.3±20.0 | 32.4±14.6 | 0.508 |  |  |
| T-P (g/dl) | 6.1±1.1 | 7.0±5.9 | 0.501 |  |  |
| Alb (g/dl) | 3.1±0.83 | 3.2±0.83 | 0.559 |  |  |
| BUN mg/dl) | 10.9±6.23 | 11.8±6.32 | 0.502 |  |  |
| Cre (mg/dl) | 0.69±0.16 | 0.85±0.96 | 0.438 |  |  |
| CRP (mg/dl) | 4.99±5.97 | 2.60±3.84 | 0.008 |  |  |
| CRP ≥ 1.5 mg/dl (N,%) | 19 (59.4%) | 49 (36.3%) | 0.0266 | 2.13 (0.78-5.26) | 0.148 |
| ESR (mm/hour) | 38.9±23.8 | 36.3±26.7 | 0.749 |  |  |
| PT-INR | 1.18±0.21 | 1.49±3.22 | 0.666 |  |  |
| APTT (sec) | 33.3±12.8 | 38.8±63.4 | 0.696 |  |  |
| Fib (mg/dl) | 338.1±98.7 | 380.7±116.5 | 0.295 |  |  |
| D-dimer (ng/dl) | 9.06±15.6 | 7.71±22.5 | 0.844 |  |  |
| FDP (ng/dl) | 30.0±48.1 | 23.0±54.5 | 0.727 |  |  |
| AT-III (%) | 88.1±21.5 | 91.6±19.7 | 0.639 |  |  |

BW: body weight, BMI: body mass index, IBD: inflammatory bowel disease, VTE: venous thromboembolism, UC: ulcerative colitis, CD: Crohn’s disease, EIM: extra-intestinal manifestation, DM: diabetes mellitus, HT: hypertension, DLp: dyslipidemia, CI: confidence interval, OR: odds ratio, 5-ASA: 5-amynosalicylate, TNF: tumor necrosis factor, IL: interleukin, JAK: Janus kinase, CI: confidence interval, OR: odds ratio, WBC: White blood cell count, Hb: hemoglobin, Ht: hematocrit, Plt: platelet, T-P: total protein, Alb: albumin, BUN: blood urea nitrogen, Cre: creatinine, CRP: C-reactive protein, ESR: erythrocyte sedimentation rate, PT-INR: prothrombin international rate, APTT: activated partial thrombin time, Fib: fibrinogen, FDP: fibrin degradation product, AT-III: antithrombin-III

**Table S3. Therapy for ATE.**

|  |  | Univariate analysis | |  |
| --- | --- | --- | --- | --- |
| Therapy for ATE | Total (n=48) | Severity and death (n=22) | Other (n=26) | p-value |
| Antithrombotic therapy | 31 (64.5%) | 14 (63.6%) | 17 (65.4%) | 1 |
| Fibrinolytic therapy | 8 (16.7%) | 2 (9.1%) | 6 (23.1%) | 0.429 |
| Catheter intervention | 12 (25.0%) | 12 (54.5%) | 0 (0%) | <0.0001 |
| Surgery | 4 (8.3%) | 4 (18.2%) | 0 % | 0.032 |
| Observation and no therapy | 0 (0%) | 0 (0%) | 0 (0%) | 1 |
| Hemorrhagic complication due to antithrombotic or fibrinolytic therapy | 2 (4.2%) | 2 (9.1%) | 0 (0%) | 0.174 |

**Table S4. The outcomes after developing ATE.**

| Days until a serious condition developed from onset of ATE (days) | 1.1±3.1 |
| --- | --- |
| Days until death from onset of ATE (days) | 0-320 |

| Outcome of therapy for ATE | Total (n=48) | Severity and death (n=22) | Other (n=26) |
| --- | --- | --- | --- |
| Improvement | 29 (60.4%) | 9 (40.9%) | 20 (76.9%) |
| retention of sequelae | 10 (20.8%) | 7 (31.8%) | 3 (11.5%) |
| Death | 2 (4.2%) | 2 (9.1%) | 0 (0%) |
| Uncertain | 7 (14.6%) | 4 (18.2%) | 3 (8.3%) |

**Table S5. Therapy for VTE.**

|  |  | Univariate analysis | |  |
| --- | --- | --- | --- | --- |
| Therapy for VTE | Total (n=167) | Severity and death (n=32) | Other (n=135) | p-value |
| Antithrombotic therapy | 140 (83.8%) | 27 (84.3%) | 113 (83.7%) | 0.583 |
| Fibrinolytic therapy | 13 (7.8%) | 6 (18.8%) | 7 (5.2%) | 0.020 |
| IVC filter | 11 (6.6%) | 11 (34.4%) | 0 ( 0%) | <0.0001 |
| Catheter intervention (other than filter) | 9 (5.4%) | 9 (28.1%) | 0 (0%) | <0.0001 |
| Surgery | 8 (4.8%) | 8 (25.0%) | 0 % | <0.0001 |
| Physical therapy | 28 (16.8%) | 5 (15.6%) | 23 (17.0%) | 1 |
| Observation and no therapy | 20 (12.0%) | 0 (0%) | 20 (15.0%) | 0.015 |
| Hemorrhagic complication due to antithrombotic or fibrinolytic therapy | 9 (5.4%) | 5 (16.1%) | 4 (3.0%) | 0.012 |
